# Droplet microfluidics-based high-throughput bacterial cultivation for validation of taxon pairs in microbial co-occurrence networks

**DOI:** 10.1038/s41598-022-23000-7

**Published:** 2022-10-28

**Authors:** Min-Zhi Jiang, Hai-Zhen Zhu, Nan Zhou, Chang Liu, Cheng-Ying Jiang, Yulin Wang, Shuang-Jiang Liu

**Affiliations:** 1 State Key Laboratory of Microbial Technology, Shandong University, Qingdao, 266000 People’s Republic of China; 2 State Key Laboratory of Microbial Resources, and Environmental Microbiology Research Center (EMRC), Institute of Microbiology, Chinese Academy of Sciences, Beijing, 100101 People’s Republic of China; 3 University of Chinese Academy of Sciences, Beijing, 100049 People’s Republic of China

**Keywords:** Microbial ecology, Ecological networks, Environmental microbiology

## Abstract

Co-occurrence networks inferred from the abundance data of microbial communities are widely applied to predict microbial interactions. However, the high workloads of bacterial isolation and the complexity of the networks themselves constrained experimental demonstrations of the predicted microbial associations and interactions. Here, we integrate droplet microfluidics and bar-coding logistics for high-throughput bacterial isolation and cultivation from environmental samples, and experimentally investigate the relationships between taxon pairs inferred from microbial co-occurrence networks. We collected *Potamogeton perfoliatus* plants (including roots) and associated sediments from Beijing Olympic Park wetland. Droplets of series diluted homogenates of wetland samples were inoculated into 126 96-well plates containing R2A and TSB media. After 10 days of cultivation, 65 plates with > 30% wells showed microbial growth were selected for the inference of microbial co-occurrence networks. We cultivated 129 bacterial isolates belonging to 15 species that could represent the zero-level OTUs (Zotus) in the inferred co-occurrence networks. The co-cultivations of bacterial isolates corresponding to the prevalent Zotus pairs in networks were performed on agar plates and in broth. Results suggested that positively associated Zotu pairs in the co-occurrence network implied complicated relations including neutralism, competition, and mutualism, depending on bacterial isolate combination and cultivation time.

Microorganisms in natural or human-engineered ecosystems interact with each other by means of mutualism, commensalism, parasitism/predation, ammensalism, and competition^1^. The sum of all microbial interactions in an ecosystem constitutes a microbial interactive network^2^. So far, the true microbial interactive networks are hardly understood, due to the microbiome complexity arising from the diverse microbial species and their interactions in the ecosystems. On the other hand, more and more microbiome data are accumulated and those data could be analyzed in silico on multiple platforms, such as Parallel-Meta Suite ^3^, Majorbio Cloud^4^. Microbial co-occurrence networks were usually reconstructed from metagenomic data or high-throughput sequencing of 16S rRNA gene amplicons from environmental and host microbiome DNA molecules^5,6^. With the information of abundance data, the microbial co-occurrence networks can be inferred using similarity-based (e.g., Pearson or Spearman correlations)^7–11^ or model-based methods (e.g., regression- and rule-based)^12–14^. For example, microbial co-occurrence networks of activated sludge from wastewater treatment plants^15^, human gut^16^, and marine environment^11,17^ were inferred from data of high-resolution and extended longitudinal and cross-sectional scales of samples. The inferred microbial co-occurrence networks were widely applied for predicting microbial interactions. But such prediction needs to be carefully examined and cautiously interpreted since most of the predictions were not experimentally demonstrated^6^. A positive relation within a co-occurrence network could be due to cross-feeding, co-colonization, niche overlap, or other random reasons^18–20^ and a negative relation could be due to competition, predation, and antagonism^21,22^.

Microbial co-occurrence networks are composed of nodes [operational taxon units (OTUs)] and edges (predicted relations among microbes)^9,23,24^. The more nodes and edges, the more complex the microbial co-occurrence networks^25,26^. The microbial co-occurrence network of a real ecosystem usually harbors hundreds of nodes and thousands of edges, which would be too complex to be experimentally demonstrated^1^. We conceived if the complex co-occurrence networks could be simplified into less complicated networks that were composed of fewer nodes and fewer edges, and if the microbes representing the nodes could be cultivated, the microbial interaction predicted from co-occurrence networks could be tested. Apparently, the simplest microbial interaction would be microbial pairs in the networks. Inspired by this conception, we designed the current study with high-throughput microfluidic microbial cultivation^27^, DNA bar-coding^28^, zero-level operational taxon units (Zotus) analysis, and similarity-based data analysis approaches^29^. We sampled the wetland ecosystem of Beijing Olympic Park (BOP). Microbial co-occurrence networks were reconstructed, prevalent Zotu pairs were identified, and extensive isolation and cultivation of microbes were performed. Zotus pairs were matched to bacterial isolates, and the microbial interactions were experimentally demonstrated.

## Methods

### Sample collection and treatments

Submerged *Potamogeton perfoliatus is the dominant plant in the sampling region of* dragon-shaped wetland ecosystem of BOP (Longitude E116°23′2.98″; Latitude N40°01′3.00″)*.* Three sites at a distance of 40 m were selected, and five *P. perfoliatus* plants, their roots, and associated sediment were collected on November 5, 2019. Samples were transferred quickly to the laboratory and were processed according to a previously reported work^30^. Briefly, the aboveground (leaves and stems) and underground parts (roots) of each *P. perfoliatus* were separated with sterile scissors. The aboveground parts were washed with 150 ml of sterilized PBS solution (containing NaCl 136 mM, Na_2_HPO_4_ 8 mM, KH_2_PO_4_ 2 mM, KCl 2.6 mM, pH 7.4) in a 50 ml sterile tube. The roots were washed into a 250 ml conical flask with 100 ml sterilized PBS (shaken at 160 rpm for 30 min, twice). To cultivate the microbial communities associated with samples, 0.5 g of aboveground parts and 0.5 g of roots were individually homogenized with 1 ml MgCl_2_ solution (10 mM) and then 5 ml sterile PBS buffer was added. Sediments (0.5 g) were suspended in 5 ml sterile PBS. The plant homogenates and sediment suspension were filtered twice using a 40-μm cell strainer (BD Falcon, USA) and diluted into 10^–1^ ~ 10^–7^ series. The plant species (*P. perfoliatus*) used in the present study was obtained from an artificial wetland and thus is not a component of wild flora. During sampling, we followed the rules of Regulations of the People's Republic of China on wild plants protection (Promulgated by Decree No. 204 of the State Council of the People's Republic of China on September 30, 1996). *P. perfoliatus* is categorized as “Least Concern” by The International Union for Conservation of Nature (IUCN), and this work did not involve any violation of IUCN Policy Statement on Research Involving Species at Risk of Extinction and the Convention on the Trade in Endangered Species of Wild Fauna and Flora.

### Microbial community inoculation and cultivation with microfluidics

The 10^–1^ ~ 10^–7^ dilution series were used for inoculation and subsequently microbial community cultivation to get microbial communities with different microbial diversities. MultiDrop Combi nL (Thermo Fisher Scientific Inc., USA) was applied to perform high throughput and automatic liquid dispensing. The microfluidic infusion tubes of the liquid dispenser were washed with 50 ml of 75% ethanol followed by sterile water. Then, 10 ml of diluted samples were pipetted into reagent bottles (50 ml centrifuge tubes). Each well of a 96-plate containing 150 μl broth was inoculated with 1 μl homogenate of the diluted sample. The treatment for each diluted sample was performed in triplicate (i.e., 3 96-well plates). The droplets of series diluted samples were respectively cultivated using R2A^31^ and Tryptone Soy Broth (TSB) (Hope Bio-Technology, China). Totally, 126 96-well plates [3 (sample types) × 7 (dilution series) × 3 (triplicate) × 2 (broth types)] were inoculated in dark condition. The inoculated plates were cultivated at 30 ℃ for 10 days. Depending on the dilutions and cell densities in samples, the numbers of wells that showed microbial growth in the 96-well plates varied. We selected those plates that more than 30% of wells showed microbial growth for further analysis. This cutoff was determined after consulting with the authors of the previously reported studies^30,32^. The biomasses in wells of retained plates (effective plates) were stored in 20% glycerol stocks (by adding 50 μl 60% glycerol) at − 80 ℃ until use.

### DNA extraction, PCR amplification, bar-coding and sequencing

The biomasses of each well in the effective plates were separately harvested and used for DNA extraction. The DNA was extracted by lysis of 6 μl of bacterial cultures in 10 μl of buffer I containing 25 mM NaOH, 0.2 mM EDTA, pH 12 and incubated at 98 °C for 30 min, and then added 10 μl of buffer II containing 40 mM Tris–HCl at pH 7.5 to lower the pH value. V4 regions of 16S rRNA genes were amplified from DNA molecules with 515F and 806R primers (Supplementary Table S1) by using 2 × Dream Taq Green PCR Master Mix (Thermo Fisher Scientific Inc., USA). The PCR was conducted in a 20 μl mixture containing 2 μl DNA template, 1 μl 10 mM of barcoded forward and reverse primers, 10 μl 2 × Dream Taq Green PCR Master Mix (Thermo Fisher Scientific Inc., USA) and 6 μl ddH_2_O under the following conditions: 94 °C for 3 min; 25 cycles of 94 °C for 30 s, 58 °C for 15 s, 72 °C for 20 s; and final elongation at 72 °C for 8 min. The PCR products were then purified with AMPure XP (Beckman Coulter GmbH, Krefeld, Germany) and then used for DNA library construction^30,32^ and sequenced on a Novaseq 6000 platform (Illumina Inc., USA) (250 bp paired-end reads) by Guangdong Magigene Biotechnology Co., Ltd, China. We adopted a two-sided barcode polymerase chain reaction (PCR) system, as described in the previously reported works^28,33^. Barcode primers used in this study are listed in Supplementary Table S1.

### Data analysis, reconstruction of co-occurrence networks, and identification of Zotu pairs

After sequence demultiplexing, 65 96-well plates with raw reads were retained for the downstream sequence analysis using UPARSE pipeline (http://drive5.com/usearch/manual/uparse_pipeline.html) with assembled paired reads (-fastq_mergepairs). Sequences were orientated by Silva 132 database(-orient)^29,34^, length-trimmed for 250 bp (-fastx_truncate), and the singleton, chimeric, and plant DNA sequences were removed. After quality control, the sequences were extracted into feature table (-otutab). Assignment for Zotu was performed according to the Silva 132 database (https://www.arb-silva.de/). As some of the prevalent Zotus for experimental validation (e.g., Zotu7) were only assigned to family level, we further annotated these sequences using the online National Center for Biotechnology Information (NCBI) BLASTn (https://blast.ncbi.nlm.nih.gov/Blast.cgi). For each 96-well plate, the Zotus showing occurrence frequencies < 30% of wells were filtered. The pairwise Spearman’s rank correlations between detected Zotus in the wells of given 96-well plates were calculated using R (version 3.6.2; https://www.r-project.org/) package psych (version 2.1.6). In addition, another tool for microbial network construction, FlashWeave (version 0.19.0)^35^, was applied to infer the co-occurrence networks using default parameters. The topological properties of co-occurrence networks were calculated using the R package igraph (version 1.2.6). To simplify the downstream experiments, we focused on the nodes with < 5 links, that is, degree centrality < 5, in these co-occurrence networks using Pajek (version 5.11)^36^ and Gephi (version 0.9.2)^37^. This cutoff could retain an average of 41.6% Zotu associations (edge numbers) (Supplementary Table S2). Zotu pairs were then identified from these co-occurrence sub-networks. To recover the robust Zotu pairs, Zotu pairs showing Spearman’s |*ρ|*> 0.6 and FDR adjusted *P* < 0.01 and occurrence frequencies > 30% were retained from the *in-silico* analysis. The occurrence frequency of a given Zotu pair was calculated as $\mathrm{FO}=\mathrm{Np}/\mathrm{Ng}$, where FO was the occurrence frequency of a given Zotu pair from different co-occurrence networks, Np was the number of times that the Zotu pair identified in the co-occurrence networks inferred by microbial communities of plates inoculated with the same medium and sample type, and Ng was the total number of co-occurrence networks constructed by microbial communities of plate inoculated with the same medium and sample type.

### Isolation of bacteria and matching the robust and prevalent Zotu pairs

To obtain bacterial isolates representing the robust and prevalent Zotu pairs in the networks, we examined 16S rRNA gene abundances in the 96-well plates and selected the wells with high abundances of targeted Zotus for bacterial isolation. The isolation was performed as follows: (1) Transferred 20 μl cell suspension from the selected well to a new sterile 1.5 ml EP tube; (2) The cell suspension was diluted into 10^–1^ ~ 10^–7^ series; (3) 100 μl of each dilution was spread onto the R2A or TSB agar plates; (4) Single colonies on the agar plates after incubation for 5 and 10 days were picked up and checked for purity by 16S rRNA gene sequencing. The taxonomy of bacterial isolates was determined following a protocol as previously described^38^. The bacterial isolates that matched the targeted Zotus were determined based on phylogenetic tree topology and 16S rRNA gene sequence similarity between the isolates and the targeted Zotus and were used for demonstration of Zotu pairs relation on agar plates or liquid broth.

### Demonstration of microbial interactions with agar plate and liquid broth

The interactions between the bacterial isolates that matched Zotu pairs were validated by the cultivation of the representative isolates on agar plates and in liquid broth. For agar plate experiments, 3 μl of the representative bacterial isolate cultures were dripped onto a TSB agar plate surface, and then 3 μl of the other matched bacterial isolate were dripped at external tangency to the firstly dripped representative isolate. The plates were cultivated at 30 ℃ for 24 h and photographs were recorded.

In addition to the experiment performed on agar plates, we also evaluated the interactions of bacterial isolates matching Zotu pairs in liquid broth. The bacterial isolates were cultivated in 5 ml TSB medium at 30 °C and 160 rpm overnight. The growing cells were harvested at 8000 rpm for 5 min and washed twice, and resuspended in sterile PBS to OD_600_ = 0.5 (Ultramicro spectrophotometer B-500Nabi; Yuanxi Instrument Co., Ltd, China). The mono- and co-culture experiments were inoculated with the same volumes (25 μl) of cell suspensions and cultured in 5 ml sterile TSB buffer. The inoculated TSB medium was cultured at 30 °C and 160 rpm. Experiments were done in triplicates. The growths were monitored with an Automated Microbiology Growth Curve Analysis System Screen C (OY Growth Curves AB Ltd., Finland).

### qPCR for quantification of *Pseudomonas *and *Aeromonas* cells in liquid cultures

The taxonomic distribution of observed prevalent Zotu pairs was mainly affiliated with *Aeromonas*. To investigate the inter-species/genus interactions, we evaluated the obtained isolates representing prevalent Zotus outside the genus *Aeromonas*. We obtained an isolate of the genus *Pseudomonas*, which is a widespread genus in environmental samples. We, therefore, focused on the co-culture experiment in liquid media for the Zotu pair representing bacterial cells from *Aeromonas* and *Pseudomonas*. Quantitative real-time PCR (qPCR) (qTOWER3/G, Analytik Jena, Germany) used SYBR Green with primer sets specifically targeting the genus *Pseudomonas* (Forward: CGTAGGTGGTTTGTTAAGTTGGATGT; Reverse: GCACCTCAGTGTCAGTATCAGT) and genus *Aeromonas* (Forward: GATTTGGAGGCTGTGTCCTTGAGAC; Reverse: AGGATTCCAGACATGTCAAGGCCA).

We amplified the DNA fragments specific to *Pseudomonas* and *Aeromonas* with the above primers by using 2 × Dream Taq Green PCR Master Mix (Thermo Fisher Scientific Inc., USA) and purified by Universal DNA Purification Kit (Tiangen Biotech Co., Ltd, China). Thus, we obtained PCR products featuring the genus *Pseudomonas* (185 bp) or the genus *Aeromonas* (186 bp). The PCR products were linked with pGM-T plasmid using pGM-T Cloning Kit (Tiangen Biotech Co., Ltd, China). The ligated DNA product was transformed into *E. coli* DH5α competent cells and plated onto LB agar plate (containing 50 μg/ml Ampicillin) at 37 ℃, overnight. The constructed plasmids were extracted using TIANprep Mini Plasmid Kit (Tiangen Biotech Co., Ltd, China), sequenced, and named *p-Pseu* and *p-Aero*, respectively. The reconstructed plasmid concentration was measured using Qubit 4.0 (Thermo Fisher Scientific, USA). The templates were diluted with sterile ddH_2_O in 8 folds (10^–1^ ~ 10^–8^ copies/ μl)^39^. The qPCR was conducted in a 20 μlL mixture containing 0.4 μlL forward and reverse primers (10 μM), 1 μlL DNA template, 10 μl ChamQ Universal SYBR qPCR Master Mix (Vazyme, Nanjing, China), 8.2 μlL ddH_2_O under the following conditions: 95 °C for 3 min; 40 cycles of 95 °C for 10 s, 60 °C for 30 s, 72 °C for 15 s, followed by the melt curve setting of 1 cycle of 95 °C for 15 s, 60 °C for 1 min and 95 °C for 15 s. The standard curve and quantitation of co-cultured bacterial isolates were amplified by using ChamQ Universal SYBR qPCR Master Mix (Vazyme, Nanjing, China) and normalized to the copy numbers of the 16S rRNA gene of each isolate (https://rrndb.umms.med.umich.edu/)*.*

## Results

### Conception of the workflow to demonstrate the microbial associations from co-occurrence networks with microbial cultivation

**Figure 1:**
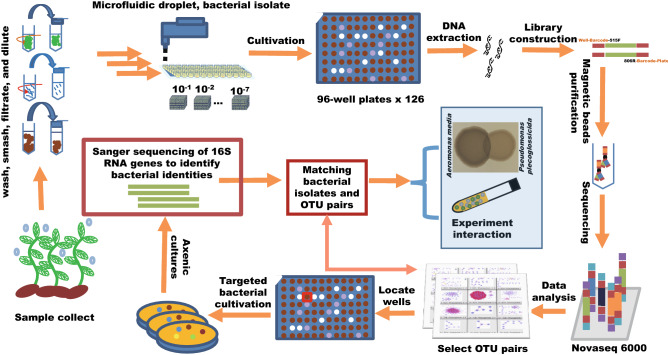
Overview of experimental demonstration of microbial interactions in co-occurrence networks. For detailed description, please refer to the method section.

Microbial co-occurrence networks are composed of nodes and edges, which usually represent microbes and statistically significant associations between microbes, respectively. We hypothesized that the microbial associations could be validated if the topological properties of networks are simplified, and if the microbes representing the nodes can be cultivated. To test this hypothesis, we designed a workflow as shown in Fig. 1. A total of 12,096 wells from 126 96-well plates were inoculated with droplets of series diluted environmental samples, wells from each 96-well plate represented the same combination of given culture condition, sample type (plants, roots, and sediments) and dilution rate (from 10^–1^ to 10^–7^). After being cultivated at 30 °C for 10 days, 69 effective (Supplementary Table S3) plates with > 30% wells showing microbial growth were retained for downstream microbial community analysis. Microbial DNA in each well was extracted, bar-coded, and sequenced for the inference of co-occurrence networks. The wells of plates showing high abundances of target Zotus were targeted for microbial isolations. Lastly, the cultivated microbial isolates were matched to Zotus in the network and used for demonstration of microbial interactions.

### Prevalent Zotu pairs in the co-occurrence networks

Depending on the microbial density in samples, the 96-well plates harbored different numbers of wells with microbial growth. We obtained 65 96-well plates (6,091 wells) that were effective with microbial growth and data analysis for co-occurrence network reconstruction. After quality control and denoise, we obtained 130 Gbp sequence data. A total of 14,377 Zotus were annotated (Supplementary Table S4). There were 217 ± 94 (average ± standard deviation) prevalent Zotus, i.e., these Zotus appeared at frequencies ≥ 30% of wells in a given 96-well plate.

**Figure 2:**
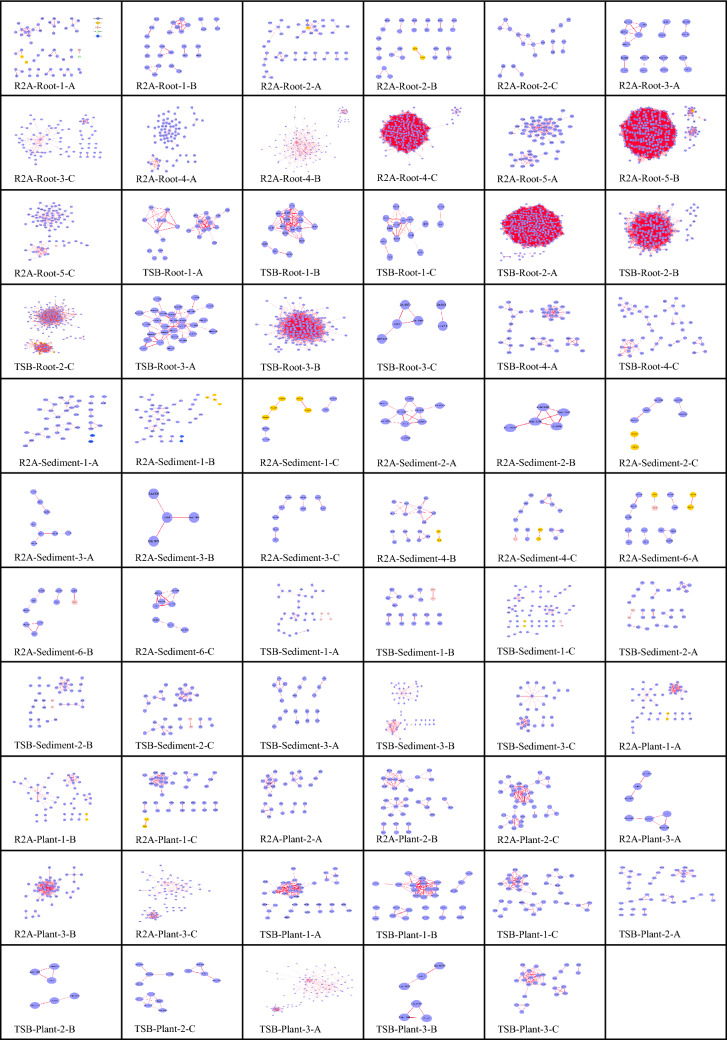
Microbial co-occurrence networks inferred based on the microbial communities of 65 inoculated 96-well plates using Spearman correlation analysis. In each panel, cascade letters and numbers are tagged to show the samples (plants for stems and leaves, roots, or sediments) into the plates, media (R2A or TSB), and the dilution level of each sample. The Letter (-A, -B, -C) represents the 3 triplicates. The dominating phyla (accounting for frequency of occurrence in 65 networks) were colored purple for Proteobacteria, pink for Firmicutes, yellow for Bacteroidetes, green for Actinobacteria, and blue for Acidobacteria. Red edges were positive interactions, while blue edges were negative interactions.

Next, we analyzed Zotus compositions and abundances in each well of the 65 plates. Accordingly, we reconstructed 65 independent microbial co-occurrence networks and further retrieved the robust (Spearman’s |*ρ|*> 0.6 and *P* < 0.01) and prevalent Zotu pairs from these microbial networks. A total of 29,805 unique Zotu pairs were identified from the 65 co-occurrence networks (Fig. 2). The Jaccard similarity matrices estimated based on edge presence-absence patterns of these sub-networks revealed that networks constructed for the same medium and dilution level of a given sample were more similar to each other than those of the other media and dilution levels (Supplementary Figure S1). As expected, the edge numbers of sub-networks decreased with the increase in dilution levels (Supplementary Figure S2). The Spearman rank correlation coefficients of Zotu pairs in the co-occurrence networks were all positive except Zotu11–Zotu12 pair from sediment sample (Supplementary Table S5), suggesting that Zotu pairs were most positively associated. Table 1 shows the top 3 prevalent Zotu pairs from co-occurrence sub-networks of samples (plants, roots, and sediments) and conditions (R2A or TSB medium). Using the 16S rRNA gene sequences, we tried to identify the phylogenetically related taxa of these Zotus, and the most closely related taxa at the genus level are listed in Table 1. Comparison between the co-occurrence networks constructed with Spearman rank correlation (Supplementary Table S5) and FlashWeave (Supplementary Table S6) revealed that an average of 51.2% Zotu pairs across the networks were shared by these two different computational methods.

**Table 1:** Top 3 prevalent Zotu pairs, their frequencies of occurrence networks and phylogenetically related taxa.

Zotu Pairs	Frequencies occurrence	Zotus and phylogenetically related Taxa/Genus		From Samples/Medium
Zotu1–Zotu15673	0.62	Zotu1	*Aeromonas*	Roots/R2A
		Zotu15673	*Aeromonas*	
Zotu18445–Zotu10	0.38	Zotu18445	*Aeromonas*	
		Zotu10	*Pseudomonas*	
Zotu8404–Zotu6	0.31	Zotu8404	*Acinetobacter*	
		Zotu6	*Acinetobacter*	
Zotu1–Zotu15673	0.81	Zotu1	*Aeromonas*	Roots/TSB
		Zotu15673	*Aeromonas*	
Zotu1–Zotu15942	0.72	Zotu1	*Aeromonas*	
		Zotu15942	*Aeromonas*	
Zotu12259–Zotu1	0.36	Zotu12259	*Aeromonas*	
		Zotu1	*Aeromonas*	
Zotu1–Zotu15673	0.57	Zotu1	*Aeromonas*	Sediments/R2A
		Zotu15673	*Aeromonas*	
Zotu1–Zotu15942	0.5	Zotu1	*Aeromonas*	
		Zotu15942	*Aeromonas*	
Zotu12707–Zotu7	0.42	Zotu12707	*Aeromonas*	
		Zotu7	*Enterobacter*	
Zotu5008–Zotu11151	0.78	Zotu5008	*Aeromonas*	Sediments/TSB
		Zotu11151	*Aeromonas*	
Zotu1–Zotu15673	0.78	Zotu1	*Aeromonas*	
		Zotu15673	*Aeromonas*	
Zotu16–Zotu46	0.78	Zotu16	*Citrobacter*	
		Zotu46	*Methylobacter*	
Zotu8404–Zotu6	0.78	Zotu8404	*Acinetobacter*	Plants/R2A
		Zotu6	*Acinetobacter*	
Zotu140–Zotu91	0.56	Zotu140	*Azorhizobium*	
		Zotu91	*Azorhizobium*	
Zotu8404–Zotu12231	0.44	Zotu8404	*Acinetobacter*	
		Zotu12231	*Aeromonas*	
Zotu1–Zotu15673	0.89	Zotu1	*Aeromonas*	Plants/TSB
		Zotu15673	*Aeromonas*	
Zotu7–Zotu11295	0.78	Zotu7	*Enterobacter*	
		Zotu11295	*Aeromonas*	
Zotu1–Zotu15942	0.67	Zotu1	*Aeromonas*	
		Zotu15942	*Aeromonas*	

The origins of those pairs (plants, roots, sediments) and the media used (R2A or TSB) for microfluidic 96-well plate cultivation are indicated.

### Cultivation of bacterial strains and matching to Zotu pairs

To experimentally demonstrate microbial interactions, it was essential to cultivate bacteria that corresponded to the Zotus from the co-occurrence networks. Based on the partial 16S rRNA genes (V4 regions) representing the Zotu pairs in Table 1, they were related to 8 bacterial genera (*Aeromonas*, *Acinetobacter*, *Citrobacter*, *Methylobacter*, *Azorhizobium*, *Enterobacter*, *Pseudomonas*, and an unidentified bacterial group). Referring to the Zotu abundances in plates, we focused on the wells and plates that showed high abundances of the Zotus in Table 1, and these wells were selected for bacterial isolation. We successfully obtained 129 bacterial strains (Supplementary Table S7) and they were phylogenetically close to 15 bacterial species based on 16S rRNA gene identities, including *Aeromonas caviae* (3 isolates), *Aeromonas hydrophila* (7 isolates), *Aeromonas media* (5 isolates), *Aeromonas rivipollensis* (17 isolates), *Elizabethkingia anopheles* (27 isolates), *Enterobacter ludwigii* (8 isolates), *Enterobacter soli* (6 isolates), *Klebsiella aerogenes* (1 isolate), *Microbacterium oxydans* (2 isolates), *Pantoea agglomerans* (18 isolates), *Pectobacterium aroidearum* (2 isolates), *Pleomorphomonas oryzae* (1 isolate), *Pleomorphomonas plecoglossicida* (10 isolates), *Pseudomonas protegens* (21 isolates), and *Raoultella ornithinolytica* (1 isolate).

**Figure 3:**
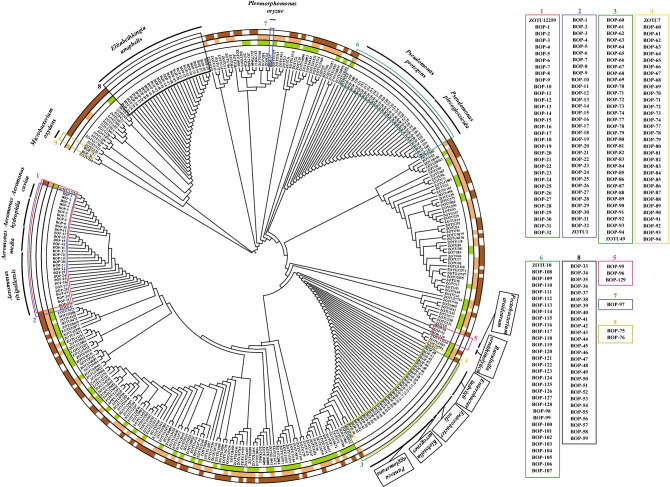
Matching bacterial isolates and Zotus from co-occurrence networks. The unscaled phylogenetic tree was constructed with neighbor-joining method based on V4 regions of bacterial 16S rRNA genes and Zotus sequences. The matched bacterial isolates and Zotus were identified according to the shortest topological distance. Inner ring: the green marked Zotus originate from plants (stems and leaves); Middle ring: the light brown marked Zotus originate from rhizospheres; Outer ring: the brown marked Zotus originate from sediments. Names of matched bacterial isolates are shown on the right side.

Next, we were trying to match the cultivated bacterial strains to the Zotus of the co-occurrence networks, and paid special attention to the Zotu pairs in Table 1. Based on the topology of the phylogenetic tree with the 16S rRNA genes of the bacterial isolates and the V4 regions of Zotus, we observed that 96 of the 129 bacterial isolates, representing 10 of the 15 bacterial species, matched 5 Zotus from co-occurrence networks. There are 32 isolated strains (i.e., BOP-1 to BOP-32) that shared identical 16S rRNA gene V4 region and clustered with Zotu 1 and Zotu12259 into the *Aeromonas* lineage in the phylogenetic tree (Fig. 3), we, therefore, randomly selected four bacterial isolates, BOP-1, BOP-5, BOP-11, and BOP-16, members of the genus *Aeromonas*, as representative isolates of either Zotu1 or Zotu12259. Similarly, BOP-61, BOP-73, BOP-74, and BOP-80 were selected from 34 bacterial strains (i.e., BOP-60 to BOP-94) sharing identical 16S rRNA gene V4 region as representative isolates of either Zotu7 or Zotu49. In addition, 20 (i.e., BOP-108 to BOP-128) and 8 bacterial strains (i.e., BOP-98 to BOP-106) that respectively shared identical 16S rRNA gene v4 region were taxonomically annotated as members of the genus *Pseudomonas* and clustered into the *Pseudomonas* lineage in Fig. 3. The isolates of BOP-102 and BOP-108 were selected as representative bacteria of Zotu10. Thirty-three of the 129 isolates, representing 5 of the 15 bacterial species, were not able to match any Zotus in Table 1. We also observed that Zotu15673, Zotu18445, Zotu8404, Zotu6, Zotu15942, Zotu12707, Zotu5008, Zotu11151, Zotu16, Zotu46, Zotu140, Zotu91, Zotu12231, Zotu11295 in Table 1 and the co-occurrence networks, respectively, did not match any cultivated bacterial isolates. However, we found that isolates BOP-61, BOP-73, BOP-74, and BOP-80 matched with Zotu7 and Zotu49 that were paired in the co-occurrence networks but not on the list of top 3 prevalent Zotu pairs.

### Bacterial associations on agar plates and in broth

**Figure 4:**
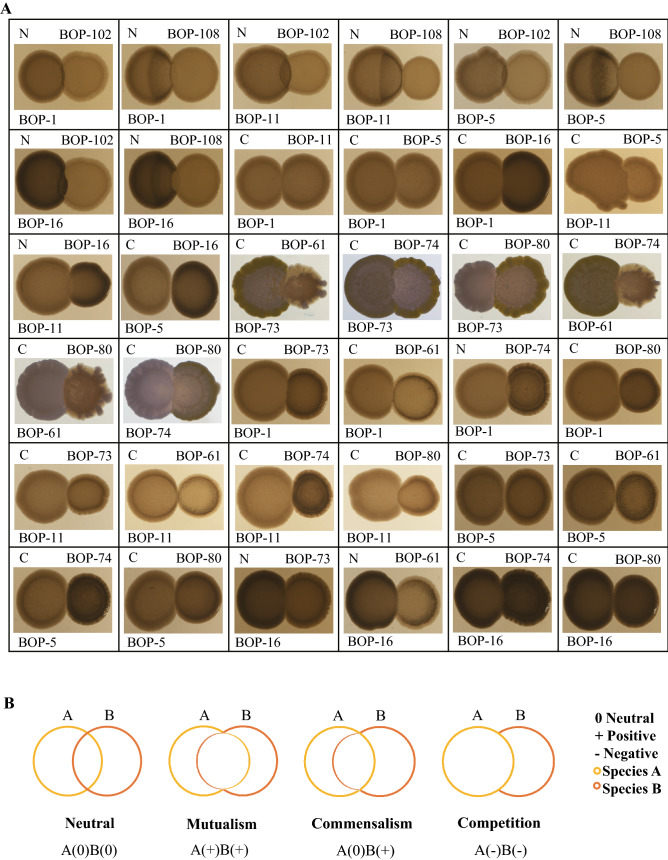
Co-cultivation experiments on TSB agar plates. (**A**) interactions of bacterial isolates on TSB agar plates. For each panel, bacterial isolate IDs were labeled at the up-right and bottom-left corners, their associations were labeled at up-left corner with letter C or N. The letters: N-neutral; C-competitive. Photographs were taken after bacterial growth on TSB agar plates for 24 h at 30 °C. (**B**) schematic diagram illustrating the morphological features of co-cultivated bacteria with different types of associations.

Using the cultivated bacterial isolates and Zotu pairs, we tried to experimentally demonstrate the microbial interactions predicted from in silico co-occurrence networking analysis. Taking all cultivated bacterial isolates and the Zotu pairs from in silico analysis into consideration, we had 36 cultivated bacterial isolate combinations that represented four Zotu pairs in the co-occurrence networks. They were 6 cultivated bacterial isolate combinations for Zotu1–Zotu12259, 6 cultivated bacterial isolate combinations for Zotu7–Zotu49, 8 cultivated bacterial isolate combinations for Zotu1–Zotu10, and 16 cultivated bacterial isolate combinations for Zotu12259–Zotu49. The selected Zotu pairs for experimental validation were confirmed by FlashWeave, except Zotu12259–Zotu49. All 36 cultivated bacterial isolate combinations were tested for their interactions on agar plates, and the results (Fig. 4) showed their mutualistic, competitive relations, or neutral (no interaction). These results suggested that positively associated Zotu pairs from co-occurrence networks implied complicated bacterial associations.

**Figure 5:**
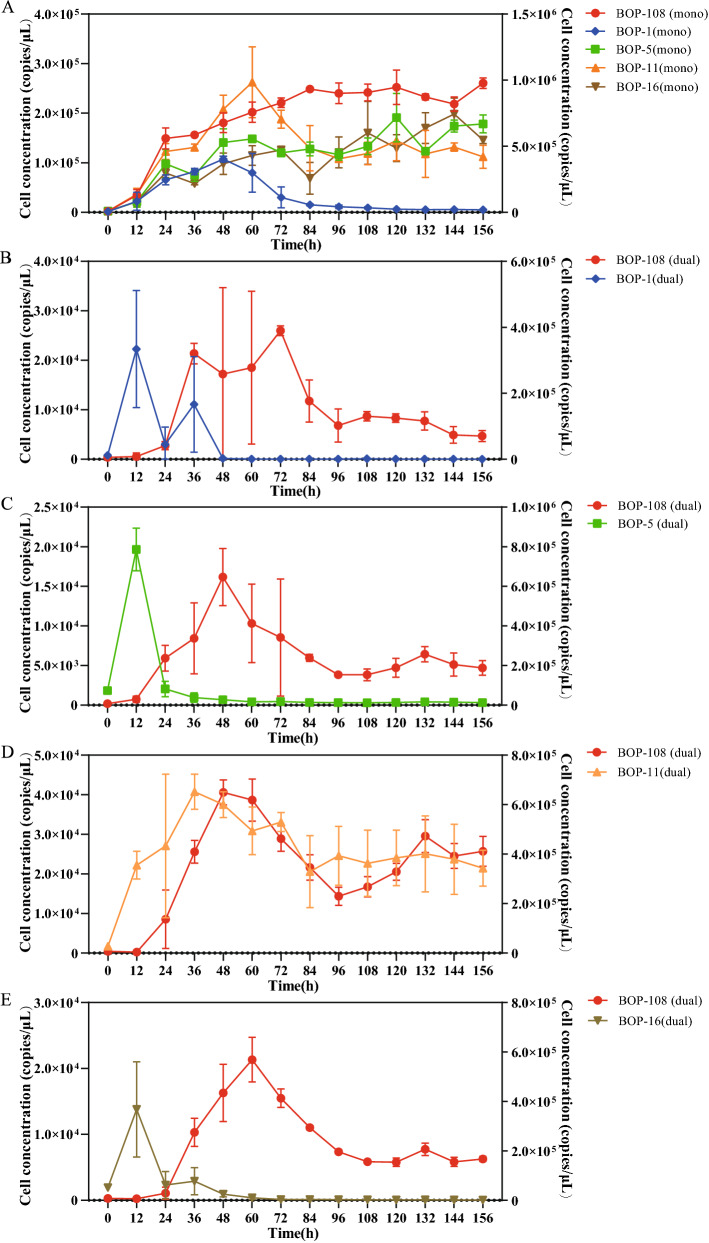
Axenic (**A**) and co-cultivation (**B–E**) of bacterial isolates BOP-1, BOP-5, BOP-11, and BOP-16 for Zotu1 and BOP-108 for Zotu10 in TSB broth. Symbols are explained in the panels. The cell densities were calculated based on 16S rRNA gene copy numbers of each isolate.

In addition to the observations on TSB agar plates, the Zotu pair of bacteria from the genera *Aeromonas* (Zotu1) and *Pseudomonas* (Zotu10) were co-cultured in broth. Given that bacterial isolates BOP-1, BOP-5, BOP-11, and BOP-16 shared identical 16S rRNA gene V4 region with Zotu1, we only selected the BOP-108 that showed a close phylogenetic relationship with Zotu10 as the representative isolate (Supplementary Figure S3). These bacterial isolates were cultivated in axenic or co-culture, and their growths (cell densities) were monitored with qPCR. Results showed that the axenic growths of bacterial isolates (Fig. 5A) were very different from co-culture. As shown in Fig. 5B, C and E, BOP-1*,* BOP-5*,* and BOP-16 for Zotu1 showed exponential growth in the first 12 h after inoculation, and then their cell densities decreased sharply. It was noteworthy that the BOP-108 for Zotu10 started growth right on 12 h after inoculation. This observation reminded us that BOP-108 for Zotu10 was possibly competitive or even inhibitive to BOP-1, BOP-5, and BOP-16 for Zotu1. The isolate BOP-11 for Zotu1 showed very differently from axenic culture when co-cultivated with BOP-108 for Zotu10 (Fig. 5D). The growth of BOP-11 and BOP-108 apparently synchronized, suggesting that they were neutral or mutual to each other.

## Discussion

Co-occurrence network analyses based on metagenomic and amplicon sequences have been frequently used to infer microbial relations in environmental or host microbiomes, and further predict microbial interactions (positive or negative) in these environments^9,23,24,40,41^, which are informatic for discovering the true microbial interactions attributing to either microbial properties (such as metabolic coupling, nutrient competition, etc.) or from environmental filtration (such as responding similarly to environmental factors or due to physical separation)^42,43^. In the present study, we established an experimental workflow for demonstrating the interactions of Zotu pairs in co-occurrence networks with bacterial isolates. The workflow consisted of high-throughput microbial cultivation with droplet microfluidics, DNA bar-coding, and sequencing, matching microbial isolates to Zotu pairs, and testing interactions of bacterial isolates on solid and in broth media. The application of dilution cultivation during high-throughput microbial cultivation with droplet microfluidics significantly simplified the microbial community compositions, which is important for the identification of robust and reliable correlations in ecosystems with extremely high microbial diversity. This workflow also took the advances of droplet microfluidics, which enables high-throughput cultivation and enumeration in an array of nanoliter droplets^44^. This droplet microfluidics also holds the biggest promise to obtain less complex microbial consortia in microliter droplets via a high-throughput manner. Although biases of selected microbial growth might be accumulated due to differences in growth rates and culture conditions (medium, temperature, aeration, etc.), thousands of droplets containing microbial sub-communities from a single sample allowed us to infer the co-occurrence networks using limited numbers of biological samples and to identify the robust associated Zotu pairs.

In this study, we applied the workflow and demonstrated the Zotu pairs from the co-occurrence network implied complicated relations in the BOP wetland ecosystem. We observed competitive and mutual relations for positively associated Zotu pairs in co-occurrence networks. Besides, most of the co-cultures of these associated Zotu pairs did not show any relations on agar plates, suggesting that microbial interactions might be more complicated in real-world ecosystems. We observed in broth culture that the bacterial isolates interacted differently from that on agar plates and the interactions changed over time (e.g., Zotu1–Zotu10), which might be attributed to the spatial structure. It is also worth noting that the isolates shared identical 16S rRNA gene V4 region with Zotu1 (affiliated with the genus *Aeromonas*) showed divergent ecological interactions with the bacterial isolate representing Zotu10 (affiliated with genus *Pseudomonas*) in broth. As reported, co-culture experiments of *Pseudomonas aeruginosa* and *Aeruginosa hydrophila* with chitin showed biphasic course, that is, grow along without affecting each other in the first phase and one (i.e., *Aeruginosa hydrophila*) subsequently inhibited by the other species (i.e., *Pseudomonas aeruginosa*) in the second phase^45,46^. This biphasic interaction was similar to our co-culture experiments between Zotu1 representative isolates (BOP-1*, Aeromonas caviae*; BOP-5, *Aeromonas hydrophila*; BOP-16, *Aeromonas media*) and Zotu10 representative isolate (BOP-108, *Pseudomonas protegens*), which might be dependent on quorum sensing-regulated production of secondary metabolites by *Pseudomonas*. In contrast to the *pyocyanin* secreted by *Pseudomonas aeruginosa*, the secondary metabolites of *Pseudomonas protegens* that inhibited co-cultured organisms might be pyoluteorin^47^. Nevertheless, the outcome of co-culture BOP11 (*Aeromonas hydrophila*) and BOP-108 (*Pseudomonas protegens*) was neutral, further complicating the interpretation of Zotu associations from co-occurrence networks.

Co-occurrence networks can be inferred from the abundances of Zotus (usually based on 16S rRNA genes)^48–51^ or microbial taxa (here refers to those based on metagenomes) or functional genes^30,32,52^. The bar-coding high-throughput amplicon sequencing enables us to decipher the microbial community compositions (Zotus) in thousands of cultivated samples in a cost-effective way. However, we found that Zotus assignment to lower taxonomic levels using 16S rRNA genes from high-throughput amplicon sequencing (i.e., V4 or V3V4 regions of 16S rRNA genes)^43–46^ was not ideal for the established workflow. One Zotu might be a sum of several bacterial strains sharing a high identity of V4 regions^53^, and this happened to the Zotu1 and Zotu49 which each matched four bacterial isolates (Fig. 3). Taking Zotu49 as an example, the different combinations for cultivated bacterial isolates showed inconsistent interactions on agar plates. The fact that one Zotu matched more than one bacterial isolate complicated experimental design and significantly increased the workload of experimental demonstration. Due to the limited resolution of the 16S rRNA gene V4 region for taxon assignments, it is doubtful whether Zotu picked from amplicon sequences could be considered an effective taxonomic unit for the experimental validation of microbial interactions in our proposed workflow. Nevertheless, we believe whole genomic information from metagenomic data (such as MAGs) would increase the resolution for taxon assignments and would improve our workflow, and we are currently working on this idea with different microbial ecosystems.

For this workflow, it should be noted that the transfer of microorganisms from the original ecosystem to plates with different media might introduce strain loss and cultivation bias. Besides, bacterial isolation and cultivation are of profound critical importance for this workflow. The limited bacterial isolates representing the Zotus in co-occurrence networks further induced the low resolution of this workflow. With the advances in high throughput cultivation protocol, it will further improve the efficiency of this workflow and provide high-resolution insights into microbial interactions between taxon pairs in co-occurrence networks. We provided experimental validation of the binary associations in the co-occurrence networks. However, microorganisms in environments engage in both direct and higher-order interactions, suggesting that the interactions between two organisms may be modulated by other species^54^. The experimental validation for higher-order interactions should be addressed in the future works.

## Conclusion

A workflow for demonstration with cultivated bacterial strains of microbial associations from co-occurrence networks has been established and was applied for the investigation of BOP wetland ecosystem. Results demonstrated that positively associated Zotu pairs displayed competitive and mutual relations, and most of the outcomes of Zotu pairs were neutral, suggesting that the microbial interactions behind the Zotu pairs in the co-occurrence networks were complicated. This study supports the conception that experimental demonstration is indispensable for interpreting microbial association and provided an example of how to carry out such a demonstration. Limitations of the current study are discussed, and further improvement of the workflow is proposed.

## Supplementary Information

Supplementary Information 1.

Supplementary Information 2.

## Data Availability

All 16S rRNA gene sequences of Zotus are available in supplementary Table S4.

## References

[1] Faust K, Raes J (2012). Microbial interactions: from networks to models. Nat. Rev. Microbiol..

[2] Ings, T. C. & Hawes, J. E. *The history of ecological networks* (ed. Dáttilo, W. & Rico-Gray, V) 15–28 (Springer, 2018).

[3] Chen Y (2022). Parallel-Meta Suite: Interactive and rapid microbiome data analysis on multiple platforms. IMeta.

[4] Ren Yi (2022). Majorbio Cloud: A one-stop, comprehensive bioinformatic platform for multiomics analyses. IMeta.

[5] Liu X (2022). Distinct co-occurrence relationships and assembly processes of active methane-oxidizing bacterial communities between paddy and natural wetlands of northeast China. Front. Microbiol..

[6] Goberna M, Verdú M (2022). Cautionary notes on the use of co-occurrence networks in soil ecology. Soil Biol. Biochem..

[7] Barberán A, Bates ST, Casamayor EO, Fierer N (2012). Using network analysis to explore co-occurrence patterns in soil microbial communities. ISME J.

[8] Zhou J (2010). Functional molecular ecological networks. MBio.

[9] Xun W (2021). Specialized metabolic functions of keystone taxa sustain soil microbiome stability. Microbiome.

[10] Liu F (2021). Cable bacteria extend the impacts of elevated dissolved oxygen into anoxic sediments. ISME J..

[11] Gao P (2020). Influences of seasonal monsoons on the taxonomic composition and diversity of bacterial community in the eastern tropical Indian Ocean. Front. Microbiol..

[12] Tsai KN, Lin SH, Liu WC, Wang DY (2015). Inferring microbial interaction network from microbiome data using RMN algorithm. BMC Syst. Biol..

[13] Kurtz ZD (2015). Sparse and Compositionally robust inference of microbial ecological networks. PLoS Comput. Biol..

[14] Steinway SN, Biggs MB, Loughran TP, Papin JA, Albert R (2015). Inference of network dynamics and metabolic interactions in the gut microbiome. PLoS Comput. Biol..

[15] Werner JJ (2011). Bacterial community structures are unique and resilient in full-scale bioenergy systems. Proc. Natl. Acad. Sci. U. S. A..

[16] Halfvarson J (2017). Dynamics of the human gut microbiome in inflammatory bowel disease. Nat. Microbiol..

[17] Milici M (2016). Co-occurrence analysis of microbial taxa in the Atlantic ocean reveals high connectivity in the free-living bacterioplankton. Front. Microbiol..

[18] Woyke T (2006). Symbiosis insights through metagenomic analysis of a microbial consortium. Nature.

[19] Ribeck N, Lenski RE (2015). Modeling and quantifying frequency-dependent fitness in microbial populations with cross-feeding interactions. Evolution.

[20] Wang X (2020). Niche differentiation of comammox *Nitrospira* in the mudflat and reclaimed agricultural soils along the north branch of Yangtze river estuary. Front. Microbiol..

[21] Williams RJ, Howe A, Hofmockel KS (2014). Demonstrating microbial co-occurrence pattern analyses within and between ecosystems. Front. Microbiol..

[22] Frey-Klett P (2011). Bacterial-fungal interactions: hyphens between agricultural, clinical, environmental, and food microbiologists. Microbiol. Mol. Biol. Rev..

[23] Forster D (2021). Lake ecosystem robustness and resilience inferred from a climate-stressed protistan Plankton network. Microorganisms.

[24] Brandon-Mong GJ, Shaw GTW, Chen WH, Chen CC, Wang DR (2020). A network approach to investigating the key microbes and stability of gut microbial communities in a mouse neuropathic pain model. BMC Microbiol..

[25] Guimerà R, Nunes Amaral LA (2005). Functional cartography of complex metabolic networks. Nature.

[26] Guimera R, Amaral LA (2005). Cartography of complex networks: modules and universal roles. J. Stat. Mech..

[27] Jiang CY (2016). High-throughput single-cell cultivation on microfluidic streak plates. Appl. Environ. Microbiol..

[28] Zhang J (2021). High-throughput cultivation and identification of bacteria from the plant root microbiota. Nat. Protoc..

[29] Edgar RC (2010). Search and clustering orders of magnitude faster than BLAST. Bioinformatics.

[30] Bai Y (2015). Functional overlap of the *Arabidopsis* leaf and root microbiota. Nature.

[31] Reasoner DJ, Geldreich EE (1985). A new medium for the enumeration and subculture of bacteria from potable water. Appl. Environ. Microbiol..

[32] Zhang JY (2019). NRT1.1B is associated with root microbiota composition and nitrogen use in field-grown rice. Nat. Biotechnol..

[33] Walters WA (2011). PrimerProspector: de novo design and taxonomic analysis of barcoded polymerase chain reaction primers. Bioinformatics.

[34] Quast C (2013). The SILVA ribosomal RNA gene database project: improved data processing and web-based tools. Nucleic Acids Res..

[35] Tackmann J, Matias Rodrigues JF, von Mering C (2019). Rapid inference of direct interactions in large-scale ecological networks from heterogeneous microbial sequencing data. Cell Syst..

[36] Batagelj, V. & Mrvar, *Lecture notes in computer science* (Jünger, M. & Mutzel, P.) 77–103 (Springer, 2003).

[37] Bastian, M., Heymann, S. & Jacomy, M. in *Proceedings of the third international conference on Weblogs and Social Media, ICWSM 2009, San Jose, California, USA, May 17–20, *2009.

[38] Liu C (2021). Enlightening the taxonomy darkness of human gut microbiomes with a cultured biobank. Microbiome.

[39] Green MR, Sambrook J (2018). Constructing a standard curve for real-time polymerase chain reaction (PCR) experiments. Cold Spring Harb. Protoc..

[40] Wang L (2021). Facial skin microbiota-mediated host response to pollution stress revealed by microbiome networks of individual. mSystems.

[41] Szoboszlay M, Tebbe CC (2021). Hidden heterogeneity and co-occurrence networks of soil prokaryotic communities revealed at the scale of individual soil aggregates. Microbiologyopen.

[42] Levy R, Borenstein E (2013). Metabolic modeling of species interaction in the human microbiome elucidates community-level assembly rules. Proc. Natl. Acad. Sci. U. S. A..

[43] Liu R (2020). Bulk and active sediment prokaryotic communities in the mariana and mussau trenches. Front. Microbiol..

[44] Villa MM (2020). Interindividual variation in dietary carbohydrate metabolism by gut bacteria revealed with droplet microfluidic culture. mSystems.

[45] Jagmann N, Brachvogel HP, Philipp B (2010). Parasitic growth of *Pseudomonas aeruginosa* in co-culture with the chitinolytic bacterium *Aeromonas hydrophila*. Environ. Microbiol..

[46] Jagmann N, Philipp B (2018). SpoT-mediated regulation and amino acid prototrophy are essential for pyocyanin production during parasitic growth of *Pseudomonas aeruginosa* in a co-culture model system with *Aeromonas hydrophila*. Front. Microbiol..

[47] Ramette A (2011). *Pseudomonas protegens* sp. nov., widespread plant-protecting bacteria producing the biocontrol compounds 2,4-diacetylphloroglucinol and pyoluteorin. Syst. Appl. Microbiol..

[48] Lane DJ (1985). Rapid-determination of 16s ribosomal-rna sequences for phylogenetic analyses. Proc. Natl. Acad. Sci. U. S. A..

[49] Hamady M, Knight R (2009). Microbial community profiling for human microbiome projects: Tools, techniques, and challenges. Genome Res..

[50] Tringe SG (2005). Comparative metagenomics of microbial communities. Science.

[51] Qin JJ (2010). A human gut microbial gene catalogue established by metagenomic sequencing. Nature.

[52] Zhalnina K (2018). Dynamic root exudate chemistry and microbial substrate preferences drive patterns in rhizosphere microbial community assembly. Nat. Microbiol..

[53] Baxter NT (2019). Dynamics of human gut microbiota and short-chain fatty acids in response to dietary interventions with three fermentable fibers. mBio.

[54] Bairey E, Kelsic ED, Kishony R (2016). High-order species interactions shape ecosystem diversity. Nat. Commun..

