# Supplementary material for: Droplet microfluidics-based high-throughput bacterial cultivation for validation of taxon pairs in microbial co-occurrence networks
Source: Sci Rep. 2022 Oct 28;12:18145. doi: 10.1038/s41598-022-23000-7 (PMC9616874; doi:10.1038/s41598-022-23000-7)
Supplement: Supplementary file 1 — Supplementary Information 1. [file 41598_2022_23000_MOESM1_ESM.docx]

**Supplementary Information**

**for**

**Droplet microfluidics-based high-throughput cultivation for experimental validation of taxon pairs in microbial co-occurrence networks**

Min-Zhi Jiang^1^, Hai-Zhen Zhu ^2^, Nan Zhou ^2^, Chang Liu ^2^, Cheng-Ying Jiang^2^, Yulin Wang^1*^, Shuang-Jiang Liu^1,2,3*^

^1^State Key Laboratory of Microbial Technology, Shandong University, Qingdao 266000, P. R. China

^2^State Key Laboratory of Microbial Resources, and Environmental Microbiology Research Center (EMRC), Institute of Microbiology, Chinese Academy of Sciences, Beijing, 100101, P. R. China

^3^University of Chinese Academy of Sciences, Beijing 100049, P. R. China

*Authors for correspondence: Shuang-Jiang Liu (liusj@sdu.edu.cn); Yulin Wang (wangyulin@sdu.edu.cn)


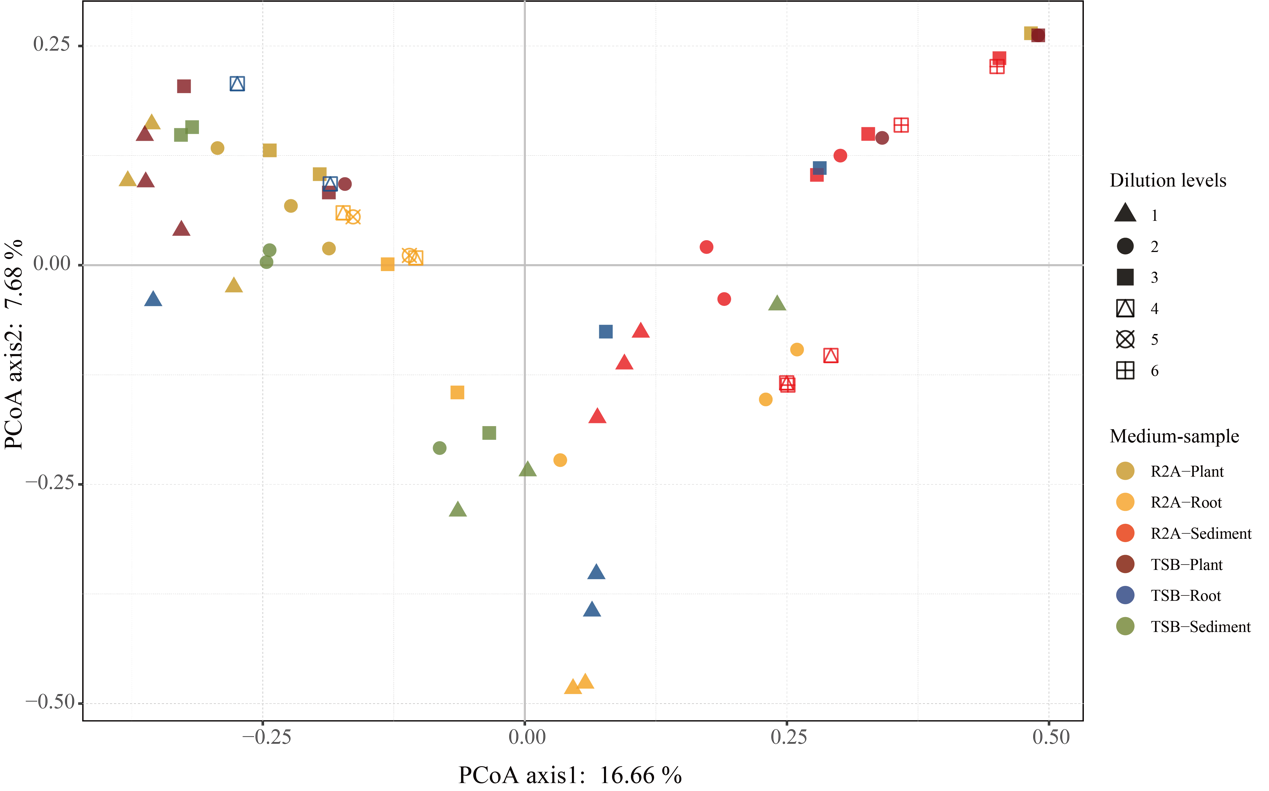


Figure S1. PCoA of the Jaccard similarity matrix estimated based on edge presence/absence in sub-networks constructed with Spearman correlation analysis.


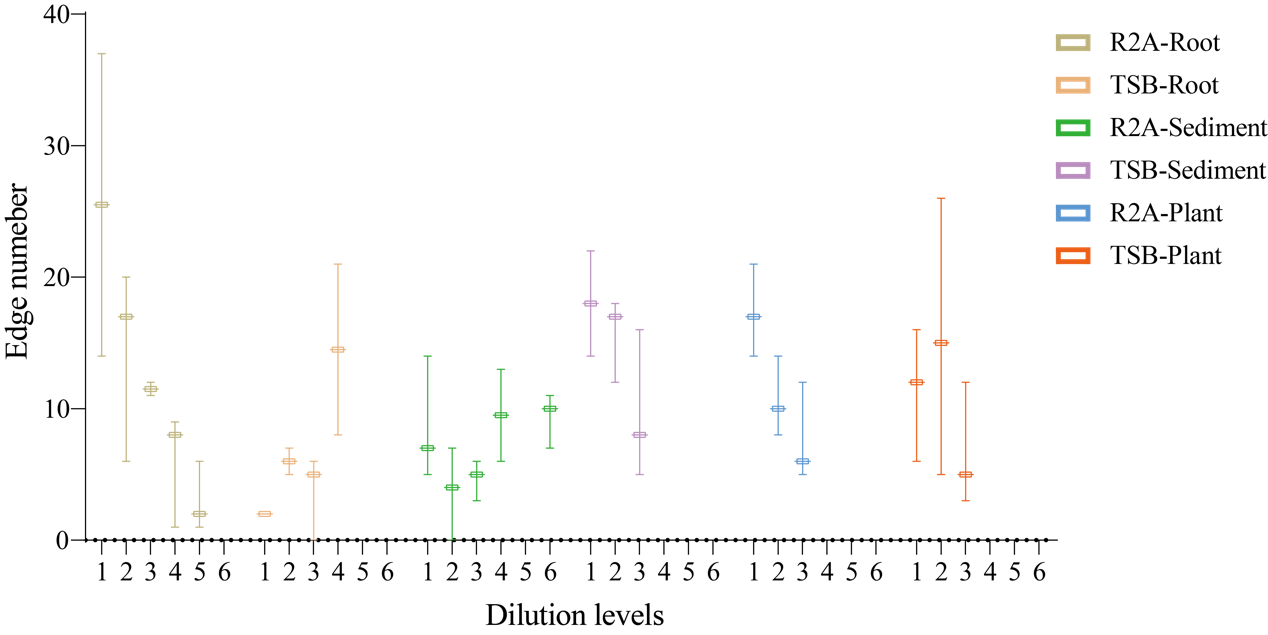


Figure S2. Edge numbers of sub-networks across different samples and cultivation media. The sub-networks used here were constructed with Spearman correlation analysis.


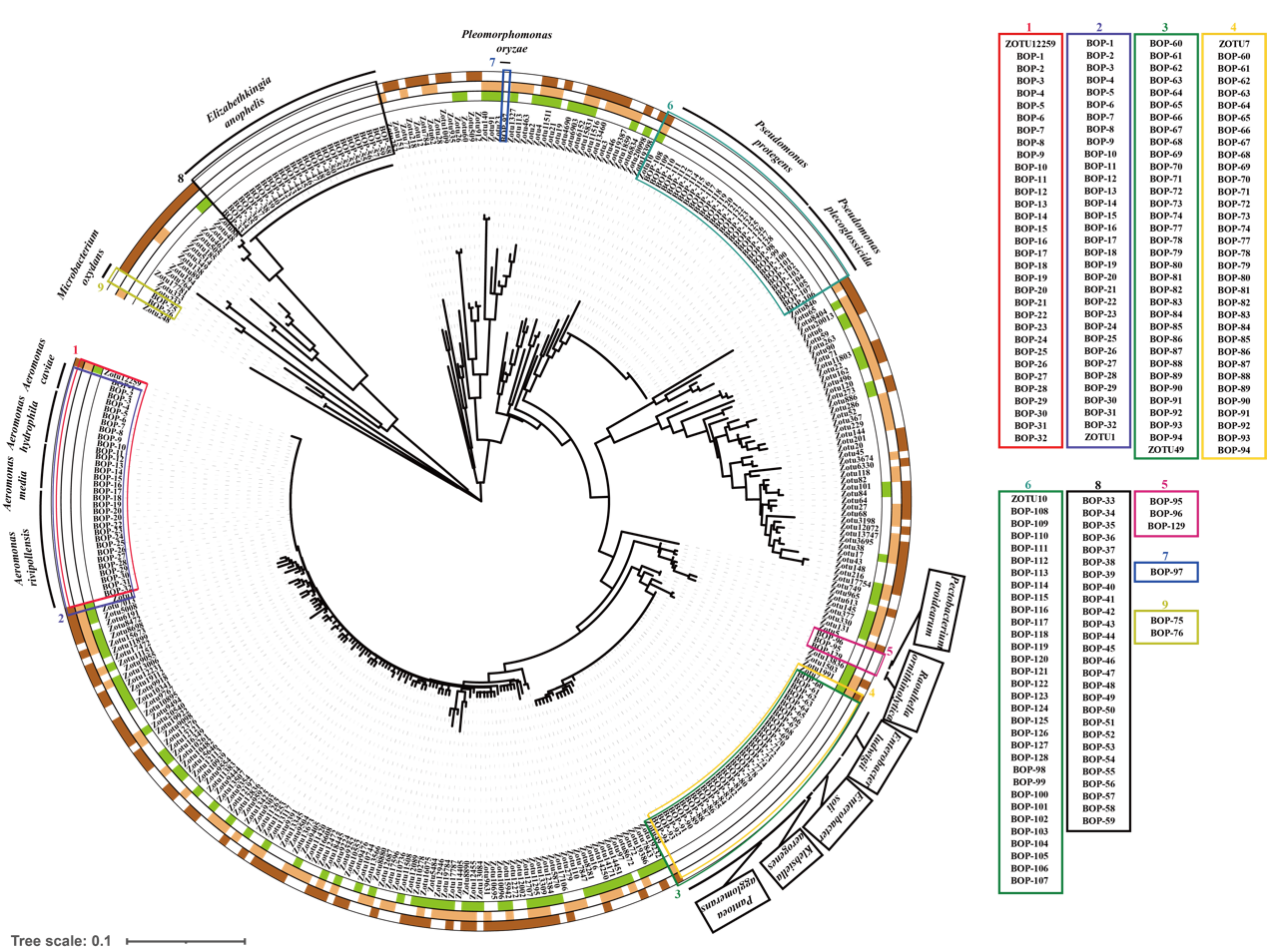


Figure S3. Matching bacterial isolates and Zotus from co-occurrence networks. The phylogenetic tree was constructed with the neighbor-joining method based on V4 regions of bacterial 16S rRNA genes and Zotus sequences. Inner ring: the green marked Zotus originate from plants (stems and leaves); Middle ring: the light brown marked Zotus originate from rhizospheres; Outer ring: the brown marked Zotus originate from sediments. Names of matched bacterial isolates are shown on the right side.
